# Supplementary material for: Comprehensive maps of escape mutations from antibodies 10-1074 and 3BNC117 for Envs from two divergent HIV strains
Source: J Virol. 2025 Apr 22;99(5):e00195-25. doi: 10.1128/jvi.00195-25 (PMC12090779; doi:10.1128/jvi.00195-25)
Supplement: Supplemental figures — Figures S1 to S7. [file jvi.00195-25-s0001.pdf]

A

	TRO11 Library A	TRO11 Library B	Combined Libraries
Total variants	86,210	97,383	183,593
Unique mutations	11,863	11,936	12,193
Percentage of all possible mutations	94.56%	95.15%	97.20%

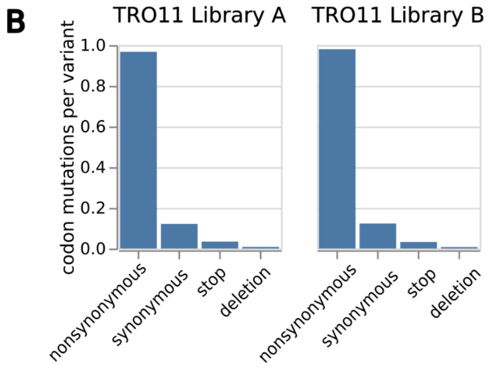

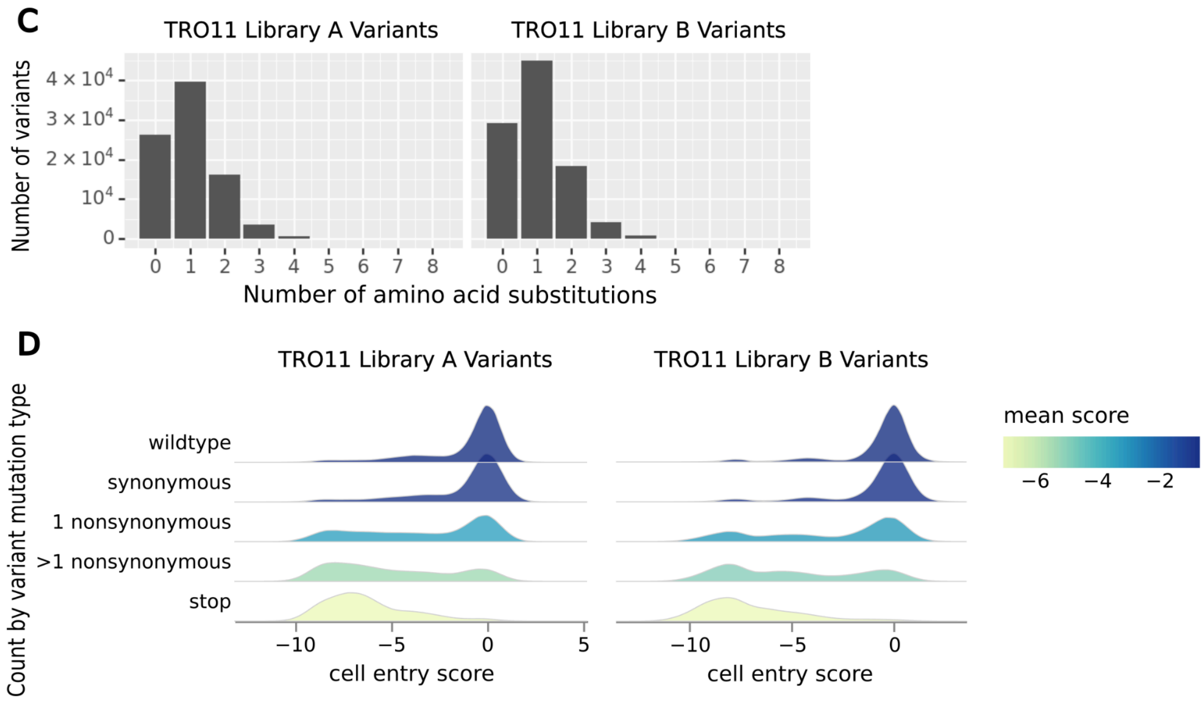

Supplemental Figure 1:

(A) Composition of variants and mutations in each individual TRO.11 Env mutant library and the combined TRO.11 libraries. (B) Average codon mutations per variant in each TRO.11 Env library, classified by type of codon mutation. (C) Distributions of the number of amino acid mutations per variant in the TRO.11 libraries. (D) Distributions of the cell entry scores of variants in each TRO.11 Env library, separated by types of codon mutations found in the mutants. Negative cell entry scores mean worse cell entry than the unmutated Env.

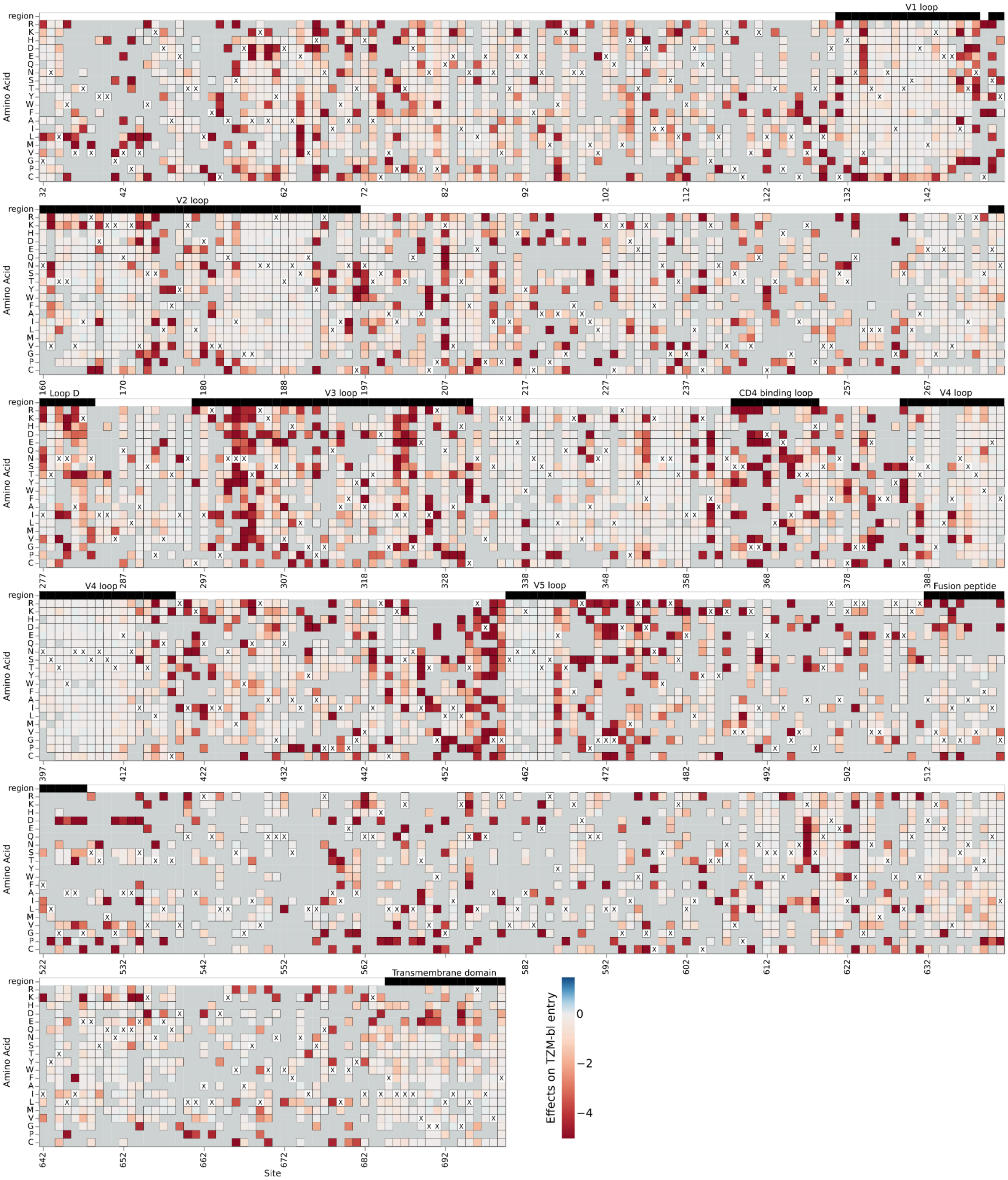

Supplemental Figure 2: Effects of mutations on BF520 Env cell entry.

For each mutation, the effect displayed in the heatmap is relative to unmutated BF520 Env. A value of zero (white) indicates no effect relative to unmutated BF520 Env, negative values (red) indicate impaired entry relative to unmutated BF520 Env, and positive values (blue) indicate improved entry relative to unmutated BF520 Env. Wildtype residues at each site are labeled with a black "X". Notable regions of Env are denoted with labeled black bars above each row. Mutations with few observations in our

experiments and therefore unconfident or no measurements are labeled with grey squares. See https://dms-vep.org/HIV_Envelope_BF520_DMS_3BNC117_10-1074/htmls/TZM-bl_entry_func_effects.html for an interactive version of this heatmap. BF520 Env deep mutational scanning measured effects of mutations on cell entry shown in this figure are previously published[26].

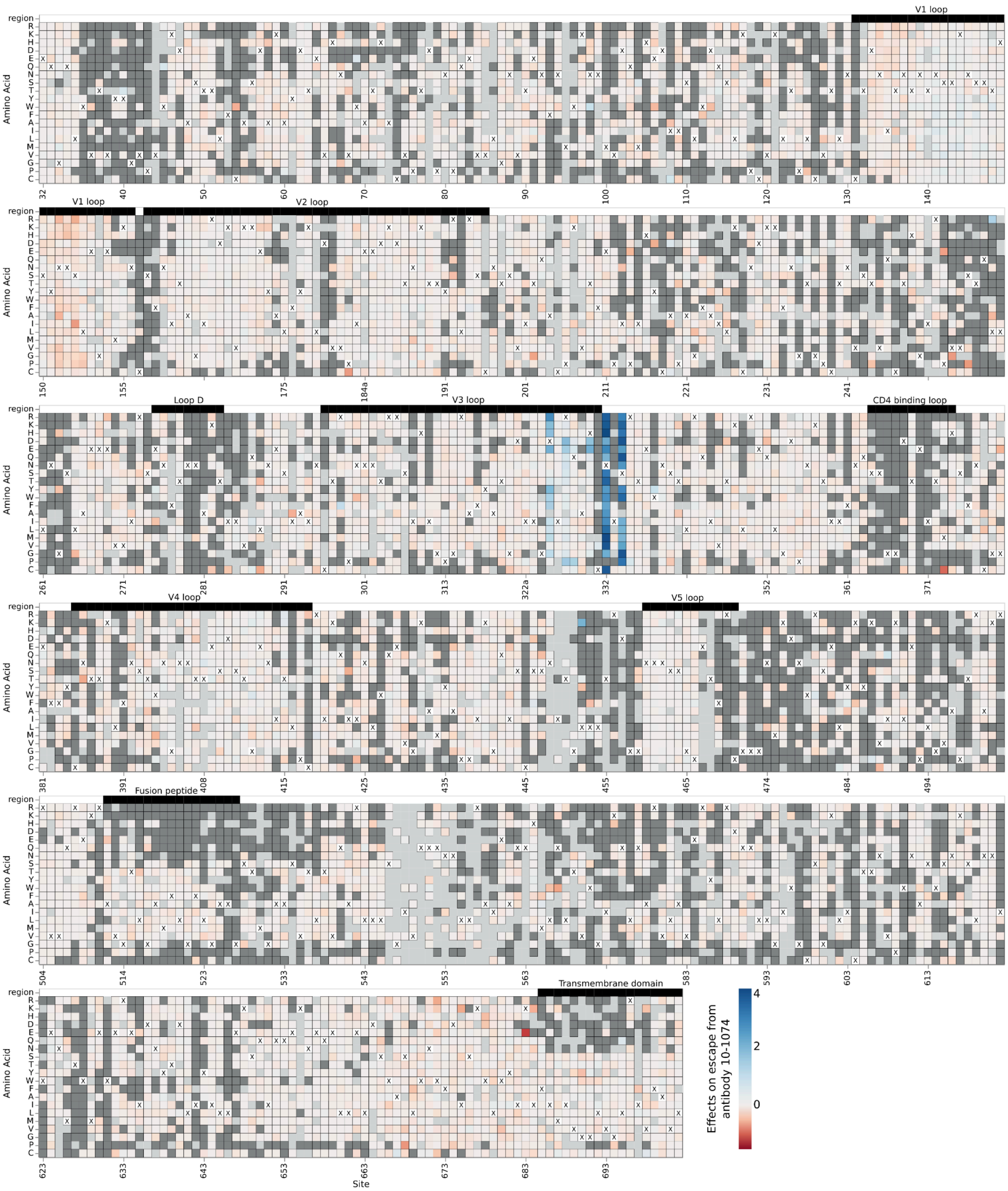

Supplemental Figure 3: Effects of mutations on TRO.11 Env escape from antibody 10-1074

A mutation effect on escape of 10-1074 of zero (white) indicates no effect relative to unmutated TRO.11 Env, positive values (blue) indicate escape from neutralization by 10-1074 relative to unmutated TRO.11 Env, and negative values (red) indicate better neutralization by 10-1074 relative to unmutated TRO.11 Env. The parental amino-acid identity at each site in TRO.11 Env is labeled with a black "X". Key regions of Env are denoted with labeled black bars above each row. Mutations that impair cell entry are colored

dark gray, and mutations with effects that were not well measured in our experiments are colored light gray. See https://dms-vep.org/HIV_Envelope_TRO11_DMS_3BNC117_10-1074/htmls/10-1074_mut_effect.html for an interactive version of this heatmap.

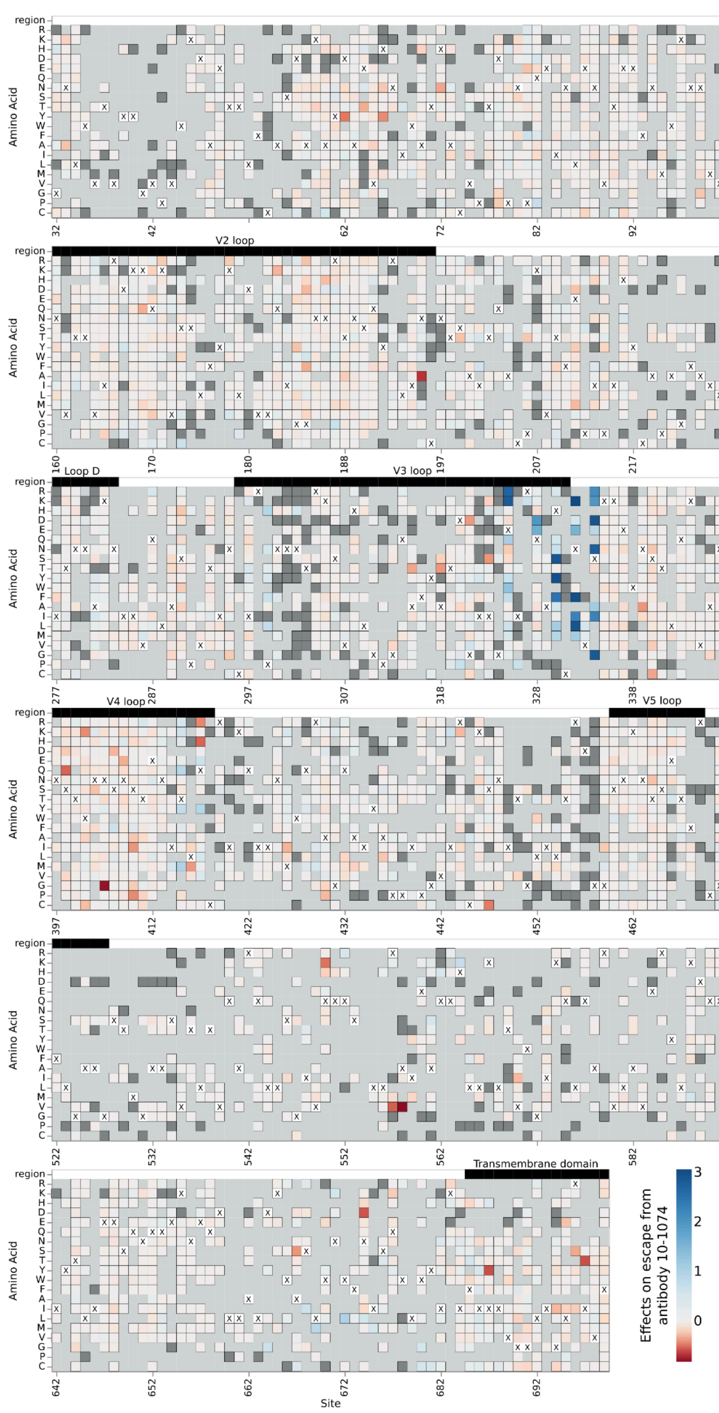

Supplemental Figure 4: Effects of mutations on BF520 Env escape from antibody 10-1074

A mutation effect on escape of 10-1074 of zero (white) indicates no effect relative to unmutated BF520 Env, positive values (blue) indicate escape from neutralization by 10-1074 relative to unmutated BF520 Env, and negative values (red) indicate higher neutralization by 10-1074 relative to unmutated BF520 Env. The parental amino-acid identity at each site in BF520 Env is labeled with a black "X". Key regions of Env

are denoted with labeled black bars above each row. Mutations that impair cell entry are colored dark gray, and mutations with effects that were not well measured in our experiments are colored light gray. See https://dms-vep.org/HIV_Envelope_BF520_DMS_3BNC117_10-1074/htmls/10-1074_mut_effect.html for an interactive version of this heatmap.

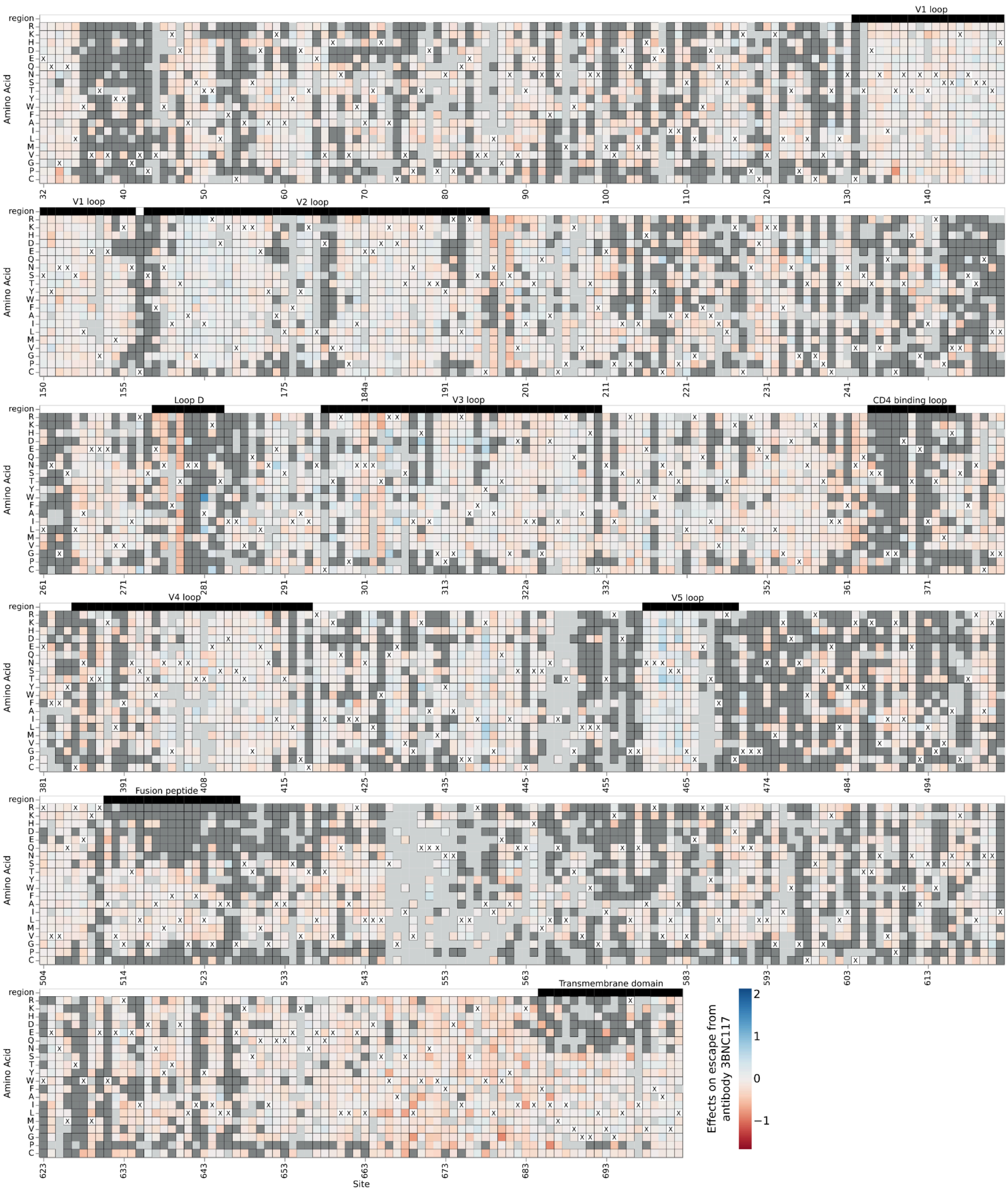

Supplemental Figure 5: Effects of mutations on TRO.11 Env escape from antibody 3BNC117

A mutation effect on escape of 3BNC117 of zero (white) indicates no effect relative to unmutated TRO.11 Env, positive values (blue) indicate escape from neutralization by 3BNC117 relative to unmutated TRO.11 Env, and negative values (red) indicate higher neutralization by 3BNC117 relative to unmutated TRO.11 Env. The parental amino-acid identity at each site in TRO.11 Env is labeled with a black "X". Key regions of Env are denoted with labeled black bars above each row. Mutations that impair cell entry are colored

dark gray, and mutations with effects that were not well measured in our experiments are colored light gray. See https://dms-vep.org/HIV_Envelope_TRO11_DMS_3BNC117_10-1074/htmls/3BNC117_mut_effect.html for an interactive version of this heatmap.

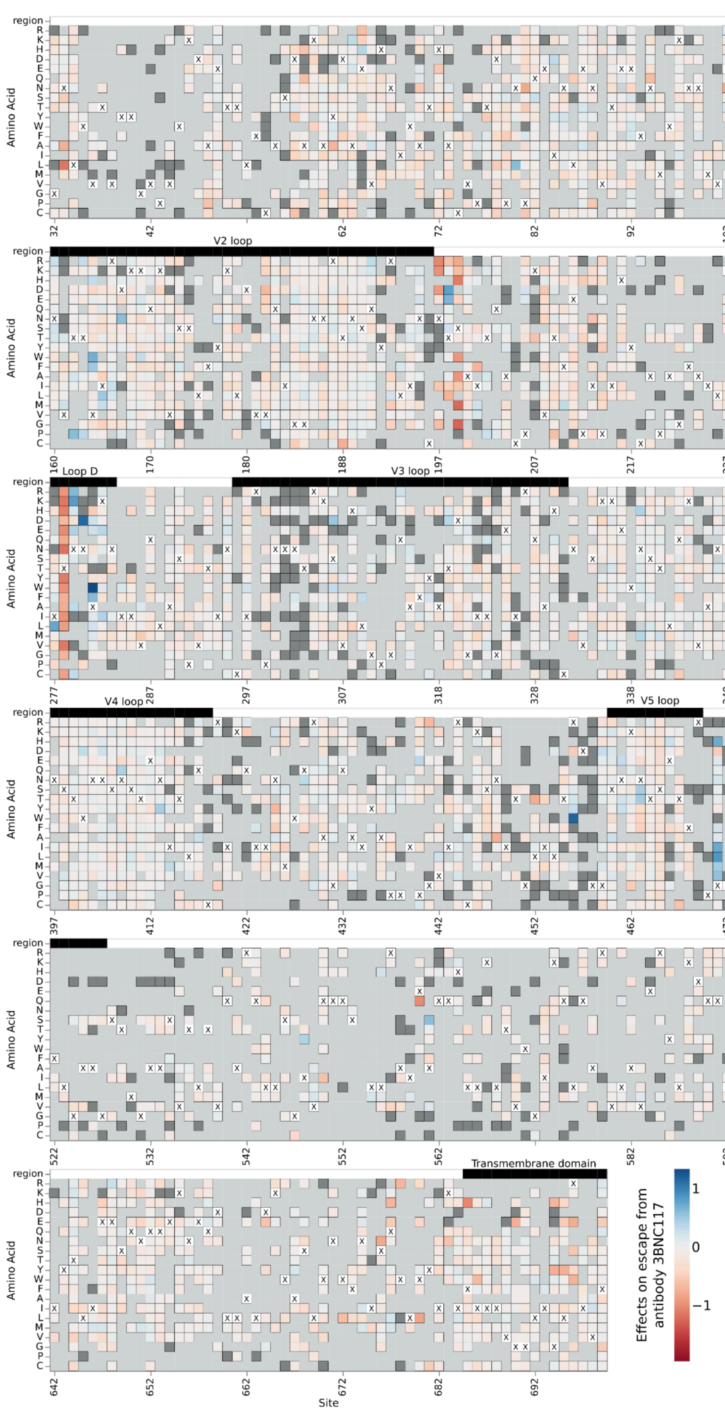

Supplemental Figure 6: Effects of mutations on BF520 Env escape from antibody 3BNC117

A mutation effect on escape of 3BNC117 of zero (white) indicates no effect relative to unmutated BF520 Env, positive values (blue) indicate escape from neutralization by 3BNC117 relative to unmutated BF520 Env, and negative values (red) indicate higher neutralization by 3BNC117 relative to unmutated BF520 Env. The parental amino-acid identity at each site in BF520 Env is labeled with a black "X". Key regions of Env are denoted with labeled black bars above each row. Mutations that impair cell entry are colored

dark gray, and mutations with effects that were not well measured in our experiments are colored light gray. See https://dms-vep.org/HIV_Envelope_BF520_DMS_3BNC117_10-1074/htmls/3BNC117_mut_effect.html for an interactive version of this heatmap. BF520 Env deep mutational scanning measured effects of mutations on escape from antibody 3BNC117 shown in this figure are previously published[26].

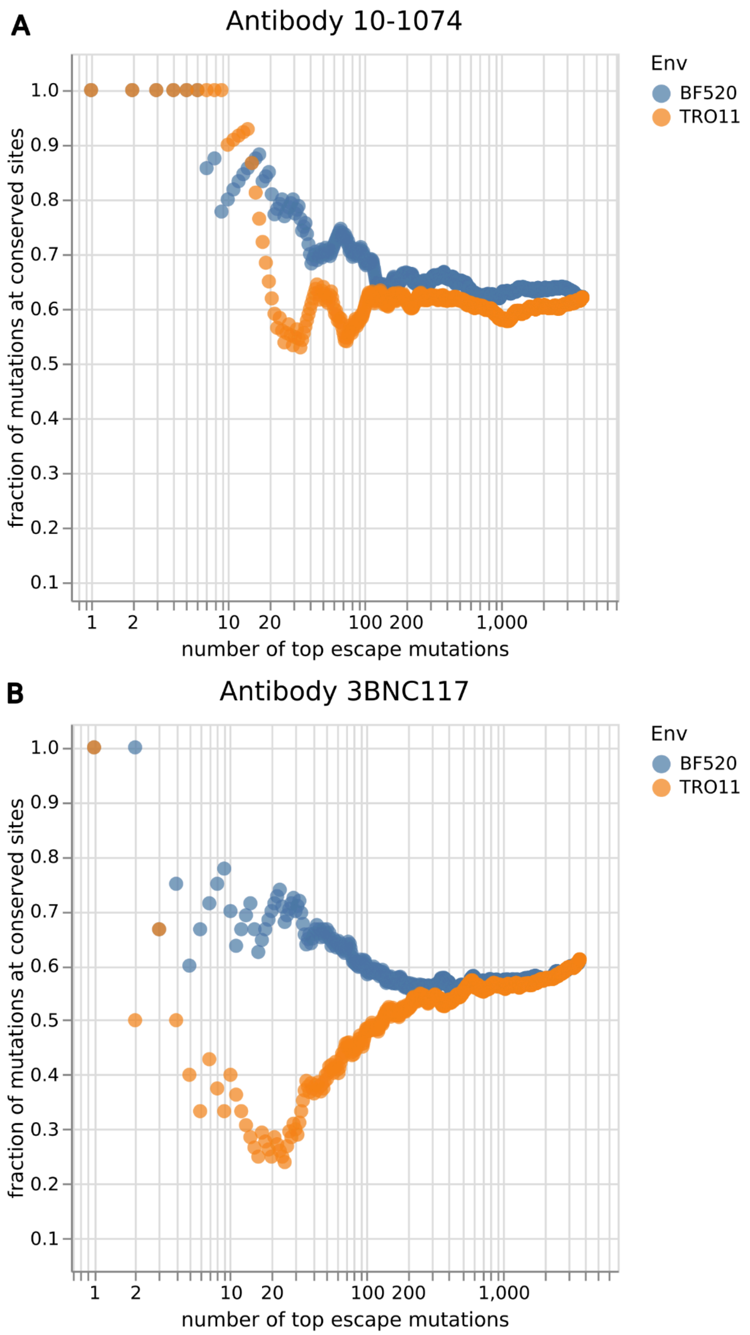

Supplemental Figure 7: Fraction of mutations at conserved sites versus number of included top escape mutations for each Env strain

Scatter plots of the fraction of mutations at sites with conserved parental amino acid residues between BF520 Env and TRO.11 Env versus the number of included top mutations sorted by mutation effect on escape from antibody 10-1074 (A) or 3BNC117 (B). Fractions at conserved sites for each number of top escape mutations were calculated independently for each Env, and points in the scatter plots are colored by Env.
